# Supplementary figures and images for: Resistance Profiling and Molecular Characterization of Extended-Spectrum/Plasmid-Mediated AmpC β-Lactamase-Producing Escherichia coli Isolated from Healthy Broiler Chickens in South Korea
Source: Microorganisms. 2020 Sep 18;8(9):1434. doi: 10.3390/microorganisms8091434 (PMC7564670; doi:10.3390/microorganisms8091434)

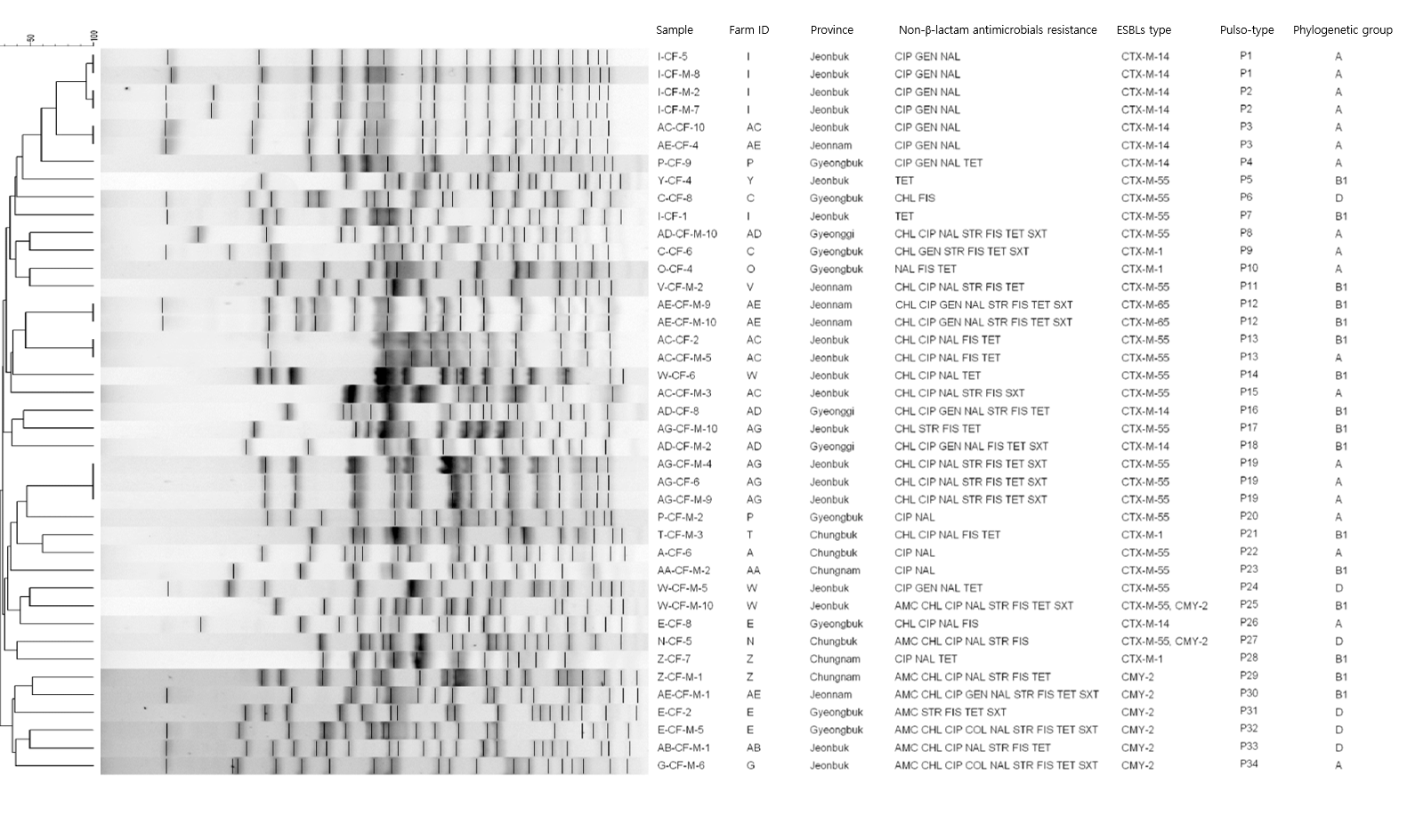

Supplement: Supplementary file 1 [file microorganisms-08-01434-s001.zip › microorganisms-934907-2nd proofed/Figure S1.tif]
