# Supplementary material for: Resistance Profiling and Molecular Characterization of Extended-Spectrum/Plasmid-Mediated AmpC β-Lactamase-Producing Escherichia coli Isolated from Healthy Broiler Chickens in South Korea
Source: Microorganisms. 2020 Sep 18;8(9):1434. doi: 10.3390/microorganisms8091434 (PMC7564670; doi:10.3390/microorganisms8091434)
Supplement: Supplementary file 1 [file microorganisms-08-01434-s001.zip › microorganisms-934907-2nd proofed/microorganisms-934907-2nd proofed-Table S1.doc]

Table S1. List of primers sequences and PCR conditions

| Primer | Sequence (5'-3') | Size (bp) | PCR condition | References |
| --- | --- | --- | --- | --- |
| CTX-M-Universal^a^ | ATGTGCAGYACCAGTAARGTKATGGC | 593 | 35 cycles; 95℃ 1min + 60℃ 1min + 72℃ 1min | [1] |
|  | TGGGTRAARTARGTSACCAGAAYCAGCGG |  |  |  |
| CTX-M-1 families^a^ | AAGACTGGGTGTGGCATTGA | 670 | 35 cycles; 95℃ 1min + 60℃ 1min + 72℃ 1min | [1] |
|  | AGGCTGGGTGAAGTAAGTGA |  |  |  |
| CTX-M-2 families^a^ | CTGGAAGCCCTGGAGAAAAG | 789 | 35 cycles; 95℃ 1min + 60℃ 1min + 72℃ 1min | [1] |
|  | TACCTCGCTCCATTTATTGC |  |  |  |
| CTX-M-8 families^a^ | GCCTGTATTTCGCTGTTG | 686 | 35 cycles; 95℃ 1min + 60℃ 1min + 72℃ 1min | [1] |
|  | TGTCATTCGTCGTACCATAA |  |  |  |
| CTX-M-9 families^a^ | GCTTTATGCGCAGACGAGTG | 703 | 35 cycles; 95℃ 1min + 60℃ 1min + 72℃ 1min | [1] |
|  | GCCAGATCACCGCAATATCA |  |  |  |
| CTX-M-1-full^a^ | CAGCGCTTTTGCCGTCTAAG | 1100 | 35 cycles; 94℃ 30sec + 55℃ 30 sec + 72℃ 1min | [2] |
|  | AAAAATGATTGAAAGGTGGT |  |  |  |
| CTX-M-9-full^a^ | GAAGCAGTCTAAATTCTTCGTGAAATAG | 1100 | 35 cycles; 94℃ 30sec + 60℃ 30 sec + 72℃ 1min | [2] |
|  | GGGCCAGTTGGTGATTTGA |  |  |  |
| *MOX^b^* | GCTGCTCAAGGAGCACAGGAT | 520 | 25 cycles; 94℃ 30 sec + 64℃ 30 sec + 72℃ 1min | [3] |
|  | CACATTGACATAGGTGTGGTGC |  |  |  |
| *CIT^b^* | TGGCCAGAACTGACAGGCAAA | 462 |  | [3] |
|  | TTTCTCCTGAACGTGGCTGGC |  |  |  |
| *DHA^b^* | AACTTTCACAGGTGTGCTGGGT | 405 |  | [3] |
|  | CCGTACGCATACTGGCTTTGC |  |  |  |
| *ACC^b^* | AACAGCCTCAGCAGCCGGTTA | 346 |  | [3] |
|  | TTCGCCGCAATCATCCCTAGC |  |  |  |
| *EBC^b^* | TCGGTAAAGCCGATGTTGCGG | 302 |  | [3] |
|  | CTTCCACTGCGGCTGCCAGTT |  |  |  |
| *FOX^b^* | AACATGGGGTATCAGGGAGATG | 190 |  | [3] |
|  | CAAAGCGCGTAACCGCATTGG |  |  |  |
| *QnrA^c^* | ATTTCTCACGCCAGGATTTG | 516 | 35 cycles; 94℃ 1min + 58℃ 1min + 72℃ 1min | [4] |
|  | GATCGGCAAAGGTTAGGTCA |  |  |  |
| *QnrB^c^* | GATCGTGAAAGCCAGAAAGG | 476 | 35 cycles; 94℃ 1min + 58℃ 1min + 72℃ 1min | [4] |
|  | ATGAGCAACGATGCCTGGTA |  |  |  |
| *QnrC^c^* | GGGTTGTACATTTATTGAATCG | 307 | 35 cycles; 94℃ 1min + 56℃ 1min + 72℃ 1min | [4] |
|  | CACCTACCCATTTATTTTCA |  |  |  |
| *QnrD^c^* | AACAAGCTGAAGCGCCTG | 580 | 35 cycles; 94℃ 1min + 56℃ 1min + 72℃ 1min | [5] |
|  | CGAGATCAATTTACGGGGAATA |  |  |  |
| *QnrS^c^* | ACTGCAAGTTCATTGAACAG | 431 | 35 cycles; 94℃ 1min + 56℃ 1min + 72℃ 1min | [5] |
|  | GATCTAAACCGTCGAGTTCG |  |  |  |
| *QnrV^c^* | GAGYTKTATGGTTTAGAYCCTCG | 71 | 25 cycles; 95℃ 56sec + 58℃ 45sec + 72℃ 15sec | [6] |
|  | TGTTCYTGYTGCCACGARCA |  |  |  |
| *QepA^c^* | GCAGGTCCAGCAGCGGGTAG | 218 | 35 cycles; 94℃ 1min + 58℃ 1min + 72℃ 1min | [7] |
|  | CTTCCTGCCCGAGTATCGTG |  |  |  |
| *acc (6’)-lb-cr^c^* | TGACCTTGCGATGCTCTATG | 508 | 35 cycles; 94℃ 30sec + 60℃ 30sec + 72℃ 30sec | [8 ] |
|  | TTAGGCATCACTGCGTGTTC |  |  |  |
| *ISEcp1^d^* | CGAGAGAAATTACACCGGTCA | 1547 | 35 cycles; 95℃ 30sec + 53℃ 30sec + 72℃ 1min | [9] |
|  | AGCCCTTCAATGCTGATGTC |  |  |  |
| *IS26 ^d^* | CTGCTTTACCAACAACATTCC | 618 | 35 cycles; 95℃ 30sec + 56℃ 30sec + 72℃ 1min | [9] |
|  | TTGTCCGGGTTGTACTCGTC |  |  |  |
| *orf477 ^d^* | TGTATGCGATGTCTGAACTG | 1238 | 35 cycles; 95℃ 30sec + 50℃ 30sec + 72℃ 1min | [10] |
|  | CTCAATAGCAGCTCGGAATA |  |  |  |
| *IS903 ^d^* | TACCGAGCTGTTCCTTGTGG | 836 | 35 cycles; 95℃ 30sec + 53℃ 30sec + 72℃ 1min | [11] |

^a^ Primer sequences used in *bla*_CTX-M_

^b^ Primer sequences used in *bla*_CMY_

^c^ Primer sequences used in plasmid mediated quinolone resistance genes

^d^ Primer sequences used in determinantion of genetic environment of *bla*_CTX-M_ and *bla*_CMY-2_

References

1. Batchelor, M.; Threlfall, E.J.; Liebana, E. Cephalosporin resistance among animal-associated *Enterobacteria*: A current perspective. *Expert Rev. Anti. Infect. Ther*. **2005**, *3*, 403-417.
2. Tamang, M.D.; Nam, H.M.; Gurung, M.; Jang, G.C.; Kim, S.R.; Jung, S.C.; Park, Y.H.; Lim, S.K. Molecular characterization of CTX-M β-lactamase and associated addiction systems in *Escherichia coli* circulating among cattle, farm workers, and the farm environment. *Appl. Environ. Microbiol*. **2013**, 79, 3898–3905.
3. Pérez-Pérez, F.J.; Hanson, N.D. Detection of plasmid-mediated AmpC beta-lactamase genes in clinical isolates by using multiplex PCR. *J. Clin. Microbiol*. **2002**, *40*, 2153–2162.
4. Tamang, M.D.; Seol, S.Y.; Oh, J.Y.; Kang, H.Y.; Lee, J.C.; Lee, Y.C.; Cho, D.T.; Kim, J. Plasmid-mediated quinolone resistance determinants *qnrA*, *qnrB*, and *qnrS* among clinical isolates of *Enterobacteriaceae* in a Korean hospital. *Antimicrob. Agents Chemother*. **2008**, *52*, 4159–4162.
5. Jacoby, G.A. AmpC β-lactamases. *Clin. Microbiol. Rev*. **2009**, *22*, 161–182.
6. Kraychete, G.B.; Botelho, L.A.B.; Campana, E.H.; Picão, R.C.; Bonelli, R.R. Updated multiplex PCR for detection of all six plasmid-mediated *qnr* gene families. *Antimicrob. Agents Chemother.* **2016**, *60*, 1416–1447.
7. Yamane, K.; Wachino, J.I.; Suzuki, S.; Kimura, K.; Shibata, N.; Kato, H.; Shibayama, K.; Konda, T.; Arakawa, Y. New plasmid-mediated fluoroquinolone efflux pump, *QepA*, found in an *Escherichia* coli clinical isolate. *Antimicrob. Agents Chemother*. **2007**, *51*, 3354–3360.
8. Kim, J.; Bae, I.K.; Jeong, S.H.; Chang, C.L.; Lee, C.H.; Lee, K. Characterization of IncF plasmids carrying the *bla*_CTX-M-14_ gene in clinical isolates of *Escherichia coli* from Korea. *J. Antimicrob. Chemother*. **2011**, *66*, 1263–1268.
9. Eckert, C.; Gautier, V.; Arlet, G. DNA sequence analysis of the genetic environment of various *bla*_CTX-M_ genes. *J. Antimicrob. Chemother*. **2006**, *57*, 14–23.
10. Saladin, M.; Cao, V.T.B.; Lambert, T.; Donay, J.L.; Herrmann, J.L.; Ould-Hocine, Z.; Verdet, C.; Delisle, F.; Philippon, A.; Arlet, G. Diversity of CTX-M β-lactamases and their promoter regions from *Enterobacteriaceae* isolated in three Parisian hospitals. *FEMS Microbiol. Lett*. **2002**, *209*, 161–168.
11. Tamang, M.D.; Gurung, M.; Kang, M.S.; Nam, H.M.; Moon, D.C.; Jang, G.C.; Jung, S.C.; Park, Y.H.; Lim, S.K. Characterization of plasmids encoding CTX-M β-lactamase and their addiction systems in *Escherichia coli* isolates from animals. *Vet. Microbiol*. **2014**, *174*, 456–462.
